# Ten Sex Talks to Boys and Ten Sex Talks to Girls (Ten Years and Older)

**Published:** 1914-10

**Authors:** 


### Book Reviews.

---

TEN SEX TALKS TO BOYS AND TEN SEX TALKS TO GIRLS (TEN YEARS AND OLDER). Irving David Steinhardt, M. D., Instructor in Clinical Surgery, and Assistant Surgeon Cornell University. J. B. Lippincott Company, June, 1914.

Just now a great deal is being said on the social question in both professional and lay papers. Much of this literature is unsuited for the purpose intended and may be productive of great harm. In these two volumes this very important question has been presented in a very commendable way. It is comprehensive enough for all purposes and it will serve admirably for a single volume book for the purposes of instruction. Sex education should be carefully guarded and care taken not to arouse morbid curiosity. These volumes are the best the author has ever reviewed on the subject and we do not hesitate to give them our full endorsement.

The author of the books takes the position that "the sexual relation is absolutely unnecessary to either sex at any time or for any reason." He does not subscribe to the view of a physiological necessity, and therefore his conclusions would be seriously opposed by many advanced physiologists. Whether the latter view is accepted or not the books afford about the most wholesome and helpful advice that could be given at the present time. This question, while important from a medical point of view, has no place in general instruction of sex to the young. The man will soon learn to adjust himself to imperative necessities, and this will be better done if he starts out with a high moral conception of the nature and purposes of sex.

